# Local Tertiary Structure Probing of Ribonucleoprotein Particles by Nuclease Fusion Proteins

**DOI:** 10.1371/journal.pone.0042449

**Published:** 2012-08-02

**Authors:** Uli Ohmayer, Jorge Perez-Fernandez, Thomas Hierlmeier, Gisela Pöll, Lydia Williams, Joachim Griesenbeck, Herbert Tschochner, Philipp Milkereit

**Affiliations:** Lehrstuhl für Biochemie III, Universität Regensburg, Regensburg, Germany; University of Lethbridge, Canada

## Abstract

Analyses of the conformational dynamics of the numerous cellular ribonucleoprotein particles (RNP) significantly contribute to the understanding of their modes of action. Here, we tested whether ribonuclease fusion proteins incorporated into RNPs can be used as molecular probes to characterize the local RNA environment of these proteins. Fusion proteins of micrococcal nuclease (MNase) with ribosomal proteins were expressed in *S. cerevisae* to produce *in vivo* recombinant ribosomes which have a ribonuclease tethered to specific sites. Activation of the MNase activity by addition of calcium led to specific rRNA cleavage events in proximity to the ribosomal binding sites of the fusion proteins. The dimensions of the RNP environment which could be probed by this approach varied with the size of the linker sequence between MNase and the fused protein. Advantages and disadvantages of the use of MNase fusion proteins for local tertiary structure probing of RNPs as well as alternative applications for this type of approach in RNP research are discussed.

## Introduction

Various techniques to study the conformational states of ribonucleoprotein particles (RNPs) are currently in use [1]–[3]. Among them are approaches measuring the accessibility of RNA for RNA modifying chemical agents or enzymes. Dependent on the specific properties of the agent chosen, this accessibility can vary in a given RNP through interactions of ribonucleotide residues with other ribonucleotides, with proteins or with ions as these interactions cause the stabilization of the different conformational states of the RNP. Through elaborated methodologies involving *in vitro* reconstitution and manipulation of RNPs, probing agents can be tethered to specific amino acid residues or ribonucleotide residues [4]–[6]. These approaches allow approximate measurements of distances between groups and of local and global conformational dynamics in the reconstituted RNP.

We wondered whether the RNA cleavage pattern produced by ribonuclease fusion proteins incorporated *in vivo* into RNPs can be used to assay characteristics of the local three dimensional RNA surrounding of these proteins. Direct RNA interaction sites of the studied protein could most likely not be mapped by such an approach. However, it could detect RNA elements which are located in tertiary structure (but not necessarily in primary structure) in proximity to the MNase fusion protein and therefore reveal characteristics of the actual folding state of the analyzed RNP (see Fig. S4 for a scheme illustrating the possible use of nuclease fusion proteins for local 3D structure probing). To avoid deleterious effects through excessive nuclease activity, the ribonucleases used for such an approach have to be tightly controlled. Micrococcal nuclease (MNase) is an enzyme secreted by micrococci showing strictly calcium dependent exo- and endoribonucleolytic and desoxyribonucleolytic activities (see for a review [7]). Its calcium dependent ribonuclease activity finds application in *in vitro* translation systems for controlled degradation of cellular mRNA and its desoxyribonuclease activity is widely used for chromatin structure probing (see for a review [8]). More recently, it was shown that local chromatin structure can be analyzed in yeast strains expressing MNase in fusion with specific chromatin associated proteins and subsequent activation of its nuclease activity by calcium addition [9], [10]. Yeast ribosomes appeared as an attractive object to test if this approach can be adapted for local 3D structure probing of RNP’s. The three dimensional structure of yeast ribosomes is well known [11] and their protein components, the ribosomal proteins, can be modified by standard genetic methodology [12], [13]. We constructed yeast strains which expressed MNase fusions of ribosomal proteins to form *in vivo* ribosomes with MNase tethered to specific ribosomal loci. Ribosomal RNA was fragmented in a calcium dependent and strain specific manner in corresponding cellular extracts. The positions of the detected cleavage sites and of the respective ribosomal proteins in the yeast ribosome structure confirmed that MNase fusion proteins can be used for local tertiary structure probing of RNPs. Further experiments indicated that the size of the linker sequences between MNase and the fused protein has impact on the dimensions of the local environment of the RNP which can be probed by MNase fusion proteins.

**Figure 1:**
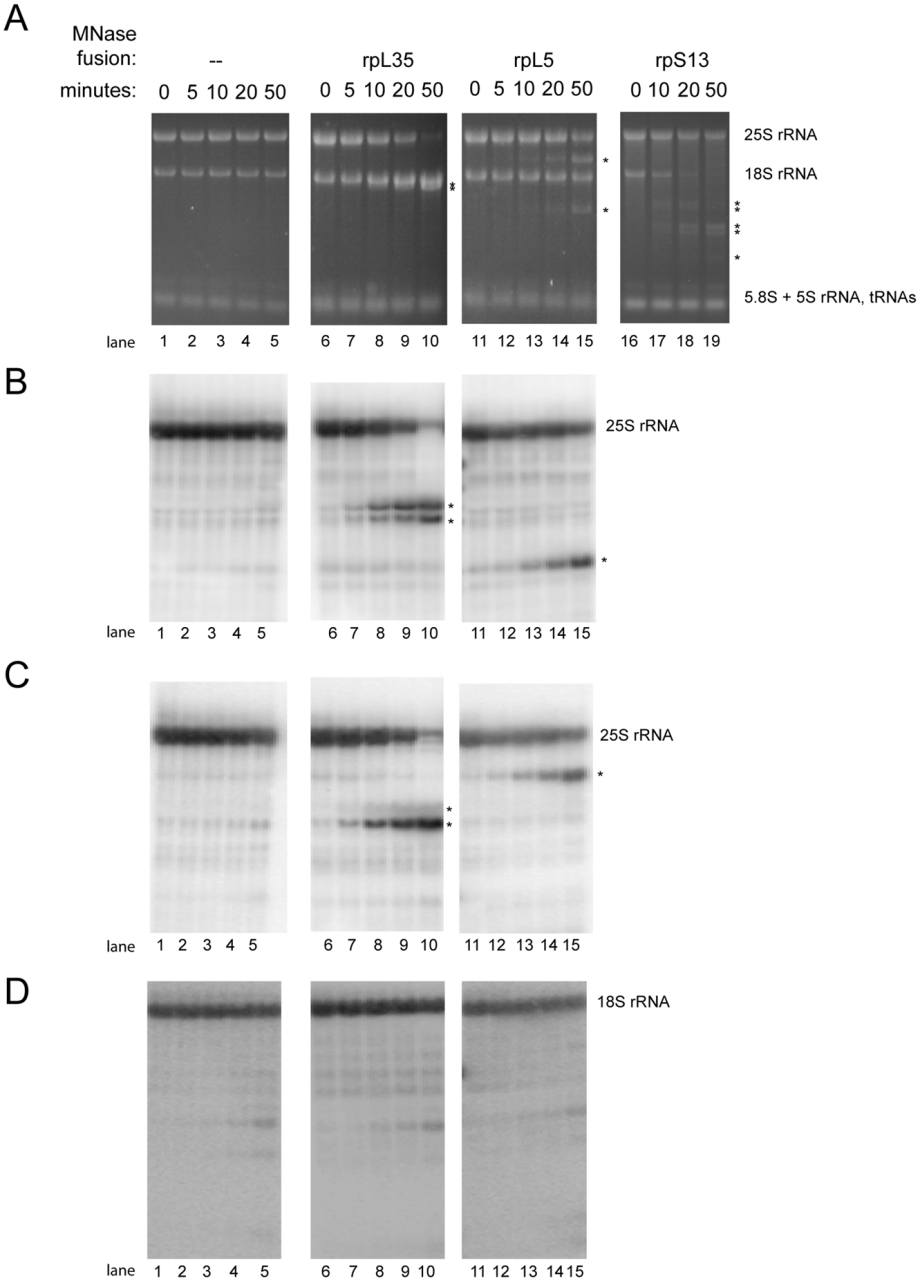
Calcium induced fragmentation of large rRNAs in extracts of cells expressing MNase fusion proteins of rpL5, rpL35 or rpS13. Cellular extracts of yeast strains expressing no MNAse fusion protein (Y206) or MNAse fusion proteins of rpL35 (Y2371), rpL5 (Y2369) or rpS13 (Y2361) were prepared as described in Materials and Methods. Samples were taken before addition of calcium chloride (0 minutes) or after incubation in the presence of calcium chloride for the indicated times at room temperature (22°C). Total RNA was extracted and separated by size on agarose gels as indicated in Materials and Methods. Gels were stained with ethidium bromide (A) or were further processed for Northern blotting (B–D) and probed with oligonucleotides detecting the 5′ end of 25S rRNA ((B), oligonucleotide O1821), the 3′ end of 25S rRNA ((C), oligonucleotide O1896) or the 3′ end of 18S rRNA ((D), oligonucleotide O1957). Positions of 25S rRNA, 18S rRNA, 5.8S rRNA, 5S rRNA and tRNAs are indicated. Stars (*) indicate positions of strain specific rRNA fragments generated during the course of extract incubation in the presence of calcium chloride.

## Results

To test for *in vivo* incorporation into yeast ribosomes and for functional complementation of ribosomal proteins expressed in fusion with MNase we made use of conditional mutant strains expressing the essential ribosomal proteins rpS13, rpL5 or rpL35 under control of the galactose inducible GAL1/10 promoter [12], [13]. Previous experiments indicated that these three proteins can be expressed in fusion with globular proteins, as the green fluorescent protein, with only minor effects on their essential functions (data not shown). In addition they are all located on distant sites of the yeast 80S ribosome [11]: rpL5 binds to the central protuberance of the 60S ribosomal subunit, while rpL35 is located on the opposite side of the large ribosomal subunit near the exit tunnel. rpS13 is a small ribosomal subunit protein which interacts with the central domain of 18S rRNA. The conditional yeast mutants were grown in galactose containing medium to support the expression of the essential ribosomal protein genes and were transformed with an empty vector or with vectors coding for the corresponding MNase fusion proteins under the control of a constitutive promoter. Similar numbers of transformants were obtained in all cases. When expression of the wildtype alleles of the ribosomal proteins was shut down on medium containing glucose as carbon source, only the transformants expressing the respective MNase fusion protein were able to grow (data not shown). Careful growth measurements of the corresponding yeast strains indicated marginal, but still detectable growth defects when solely the MNase fusion proteins of rpL5 or rpL35 were expressed on medium containing glucose as carbon source (<15% difference compared to growth of a wildtype strain, see Fig. S5). In case of rpS13, growth defects were slightly more pronounced (<40% difference compared to growth of a wildtype strain, see Fig. S5). These analyses showed that expression of the chosen fusion proteins had no general cytotoxic effects as consequence of a potential increase in cytoplasmic RNAse activities [14]. Moreover, the fusion proteins could apparently fulfill all the essential functions of the corresponding ribosomal proteins indicating their incorporation into yeast ribosomes. Consistent with this, 80S ribosomal components co-purified in a specific and efficient manner with the MNase-HA tagged fusion proteins (see Fig. S6).

**Figure 2:**
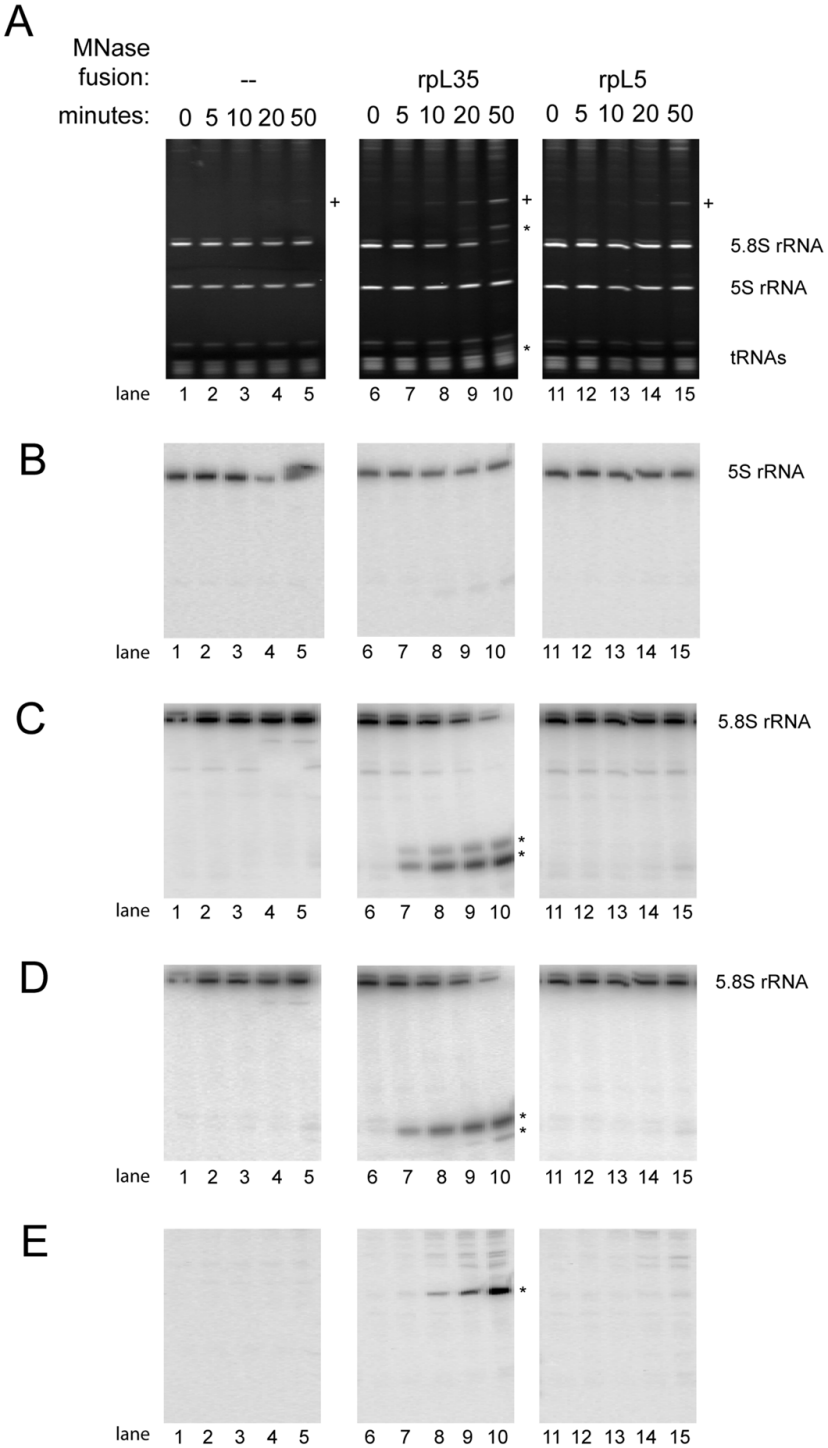
Calcium induced fragmentation of small rRNAs in extracts of cells expressing MNase fusion proteins of rpL5 or rpL35. Cellular extracts of yeast strains expressing no MNAse fusion protein (Y206) or MNAse fusion proteins of rpL35 (Y2371) or rpL5 (Y2369) were prepared as indicated in Materials and Methods. Samples were taken before addition of calcium chloride (0 minutes) or after extract incubation in the presence of calcium chloride for the indicated times at room temperature (22°C). Total RNA was extracted and separated by size on denaturing polyalcrylamide gels as described in Materials and Methods. Gels were stained with ethidium bromide (A) or were further processed for Northern blotting (B–E) and probed with oligonucleotides detecting the 5S rRNA ((B), oligonucleotide O2474), the 5′ end of 5.8S rRNA ((C), oligonucleotide O209), the 3′ end of 5.8S rRNA ((D), oligonucleotide O2959) or the 25S rRNA region 1623–1643 nucleotides downstream of the 5′ end of 25S rRNA ((E), oligonucleotide O3068). ). Positions of 5.8S rRNAs, 5S rRNA and tRNAs are indicated. Stars (*) indicate positions of strain specific rRNA fragments generated during the course of extract incubation in the presence of calcium chloride. A cross (+) marks a RNA fragment significantly detected in extracts of strains not expressing MNase fusion proteins.

Extracts were prepared of cells of these strains grown in medium containing glucose as carbon source to express solely the MNase fusions of the respective ribosomal proteins. Buffers used for extract preparation contained EGTA to quench excess of free calcium ions possibly released through breakage of subcellular compartments. Use of EGTA instead of EDTA for this purpose should preserve the concentration of free magnesium ions important for structure and function of many RNPs. Exogenous calcium was then added and extracts were incubated at 22°C. Total RNA was extracted from aliquots taken before or after addition of calcium and was analyzed by gel electrophoresis followed by ethidium bromide staining or Northern blotting (Figs. 1 and 2). As seen in Figs. 1 and 2, lanes 1–5, only minor degradation of rRNA was detected in cellular extracts prepared from a strain expressing no MNase fusion protein (see appearance of minor degradation fragment marked with (+) in Fig. 2A, lane 5). By contrast, ethidium bromide staining revealed substantial calcium induced fragmentation of rRNA in extracts from strains expressing MNase fusions of ribosomal proteins (Figs. 1A and 2A, lanes 6–19). The rRNA fragmentation patterns clearly differed for each of these strains. Fragmentation of 18S rRNA was seen in extracts of strains expressing MNase fusions of the small ribosomal subunit protein rpS13 (Fig. 1A, lanes 16–19). Fragmentation of 25S rRNA was detected in extracts of strains expressing MNase fusions of large ribosomal subunit proteins rpL5 and rpL35 (Fig. 1A, lanes 11–15 and 6–10). In addition to that, 5.8S rRNA was fragmented after addition of calcium to extracts of strains expressing MNase fusions of rpL35 (Fig. 2A, lanes 6–10). Omission of calcium during extract incubation efficiently inhibited the observed rRNA fragmentation (see Fig. S7). Addition of recombinant MNase to an extract of yeast cells expressing no MNase fusion proteins resulted in a distinct rRNA fragmentation pattern consistent with various preferred MNase cleavage sites in rRNA distributed all over the 80S ribosome (See Fig. S8). The specific rRNA fragmentation pattern observed in the extracts expressing MNase fusion proteins strongly suggested that calcium dependent nuclease activity of MNase was tethered to local ribosomal environments through its fusion with the respective ribosomal protein.

**Figure 3:**
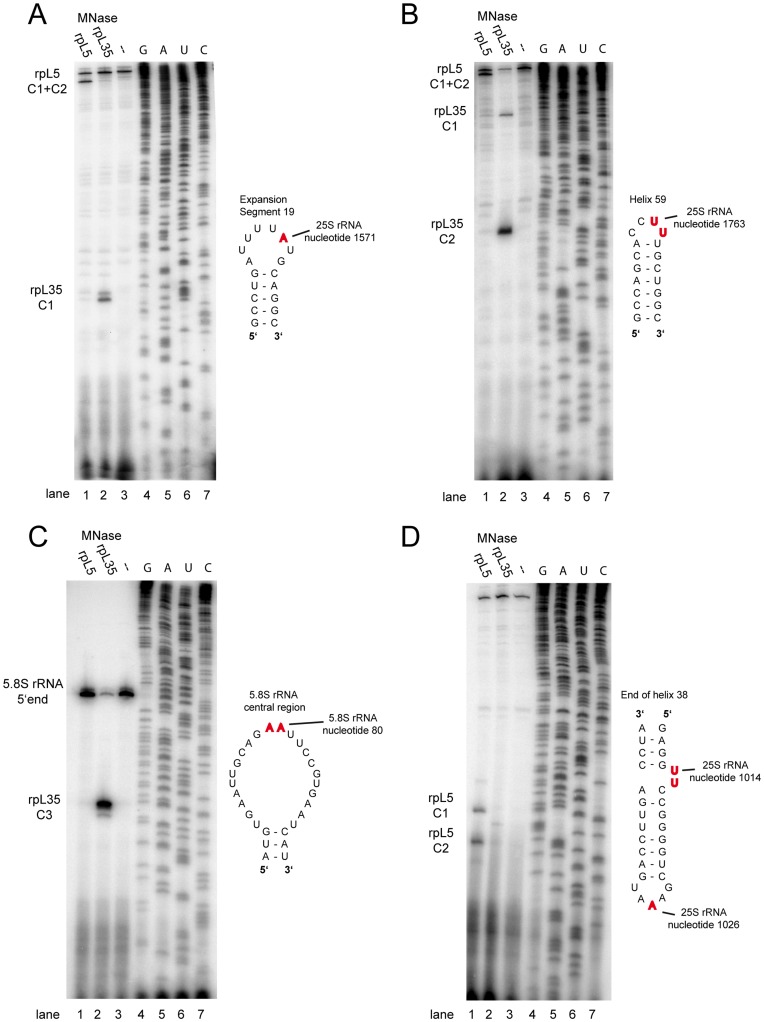
Analysis of major calcium induced rRNA 5′ ends in MNase-rpL5 or MNase-rpL35 containing ribosomes. Cellular extracts of yeast strains expressing no MNase fusion protein protein (Y206) or MNAse fusion proteins of rpL35 (Y2371) or rpL5 (Y2369) were incubated for 50 minutes at room temperature in the presence of calcium chloride. Total RNA was purified and analyzed by primer extension with primers hybridizing to a region 1623 nucleotides downstream of the 25S rRNA 5′ end ((A), oligonucleotide O3068)), a region 1855 nucleotides downstream of the 25S rRNA 5′ end ((B), oligonucleotide O1890), a region 143 nucleotides downstream of the 5.8S rRNA 5′ end ((C), oligonucleotide O2959) or a region 1068 nucleotides downstream of the 25S rRNA 5′ end ((D), oligonucleotide O3074). Primer extension products were separated by size by denaturing acrylamide gel electrophoresis and analyzed by autoradiography. Sequencing reactions of a plasmid carrying a full ribosomal DNA copy (K375) were performed in parallel (lanes 4–6 in A–D). Major detected 5′ ends are indicated on the left and were named rpL35-C1, rpL35-C2, rpL35-C3, rpL5-C1 and rpL5-C2. On the right in figures A–D secondary structure diagrams adapted from http://www.rna.ccbb.utexas.edu/ [15] of the rRNA regions of interest are shown with major detected 5′ ends highlighted in red. Helix and expansion segment numbering is according to the *E. coli* 23S rRNA helix numbering and [11].

To correlate known ribosomal protein binding sites [11] and calcium induced local RNA cleavage events in the *in vivo* formed recombinant ribosomes we aimed to characterize in more detail the sites of major nuclease actions in extracts of strains expressing MNase fusions of rpL5 and rpL35. For extracts of MNase-rpL35 expressing strains Northern blotting analyses argued for the appearance of two calcium induced cuts in the central region of the 25S rRNA (Figs. 1B and 1C, lanes 6–10) which lead to release of a small stable 25S rRNA fragment (Fig. 2E, lanes 6–10, see Fig. S9 for a map of the yeast large ribosomal subunit rRNA including positions of oligonucleotides used for Northern probing and predicted cleavage sites). At the same time, appearance of two 5.8S rRNA fragments (Figs. 2C and D, lanes 6–10, see also Fig. S9) was in good agreement with one MNase-rpL35 dependent major cut around the middle region of 5.8S rRNA. In case of MNase-rpL5 expressing strains the Northern analyses argued for major calcium dependent nucleolytic events around a region about 1000 nucleotides 3′ of the 25S rRNA 5′ end (Figs. 1B and 1C, lanes 11–15, see also Fig. S9).

**Figure 4:**
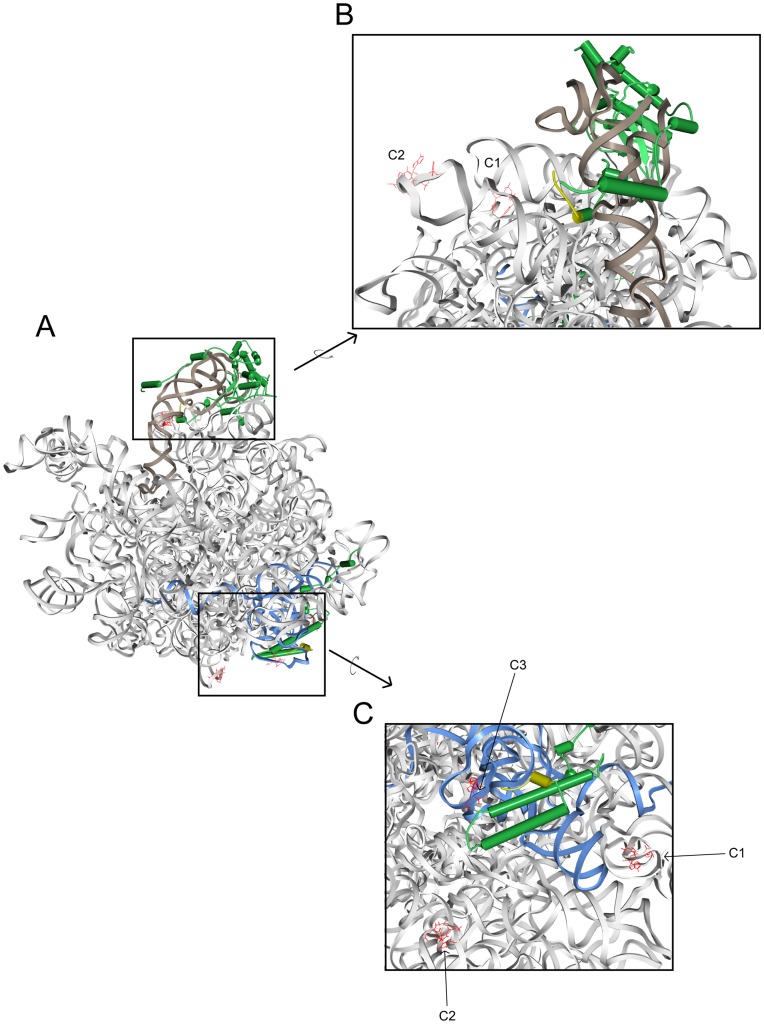
Localization of major rRNA cleavage sites in 60S ribosomal subunits containing MNase-rpL5 or MNase-rpL35. Structure of 25S rRNA (grey), 5S rRNA (brown), 5.8S rRNA (blue), rpL5 and rpL35 (green) in yeast 60S ribosomal subunits are shown in a schematic representation according to pdb files 3U5H and 3U5I [11]. The N-termini of rpL5 and rpL35 until amino acid 10 to which the MNase was fused in the corresponding strains are marked in yellow. Major detected 5′ ends are shown in a line representation and are highlighted in red. According to the nomenclature introduced in Fig. 3 they are further named C1–C3 in B and C. A) Overview of the yeast 60S ribosomal subunit with the central protuberance and rpL5 at the top and the exit tunnel and rpL35 at the bottom. B) Close up view of the central protuberance together with rpL5. C) Close up view of the region around the ribosomal exit tunnel with a high-lighted rpL35.

To map the MNase dependent 5′ ends of the resulting rRNA fragments at nucleotide resolution, primer extension analyses using corresponding primers were performed (Fig. 3). Major MNase-rpL35 dependent 5′ ends could be mapped around nucleotides 1571 and 1763 3′ of the 25S rRNA 5′ end and around nucleotide 80 3′ of the 5.8S rRNA 5′ end in extracts containing MNase-rpL35 ribosomes (Figs. 3 A, B, C, see also Fig. S9). Major MNase-rpL5 dependent 5′ ends were detected around nucleotides 1014 and 1026 3′ of the 25S rRNA 5′ end (Fig. 3D, see also Fig. S9). In agreement with a substrate preference of MNase for pyrimidine rich single stranded regions (reviewed in [4]) all four detected nucleolytic hotspots were located in such a context (see secondary structure diagrams in Figs. 3A, B, C, D, adapted from http://www.rna.ccbb.utexas.edu/, [15]).

**Figure 5:**
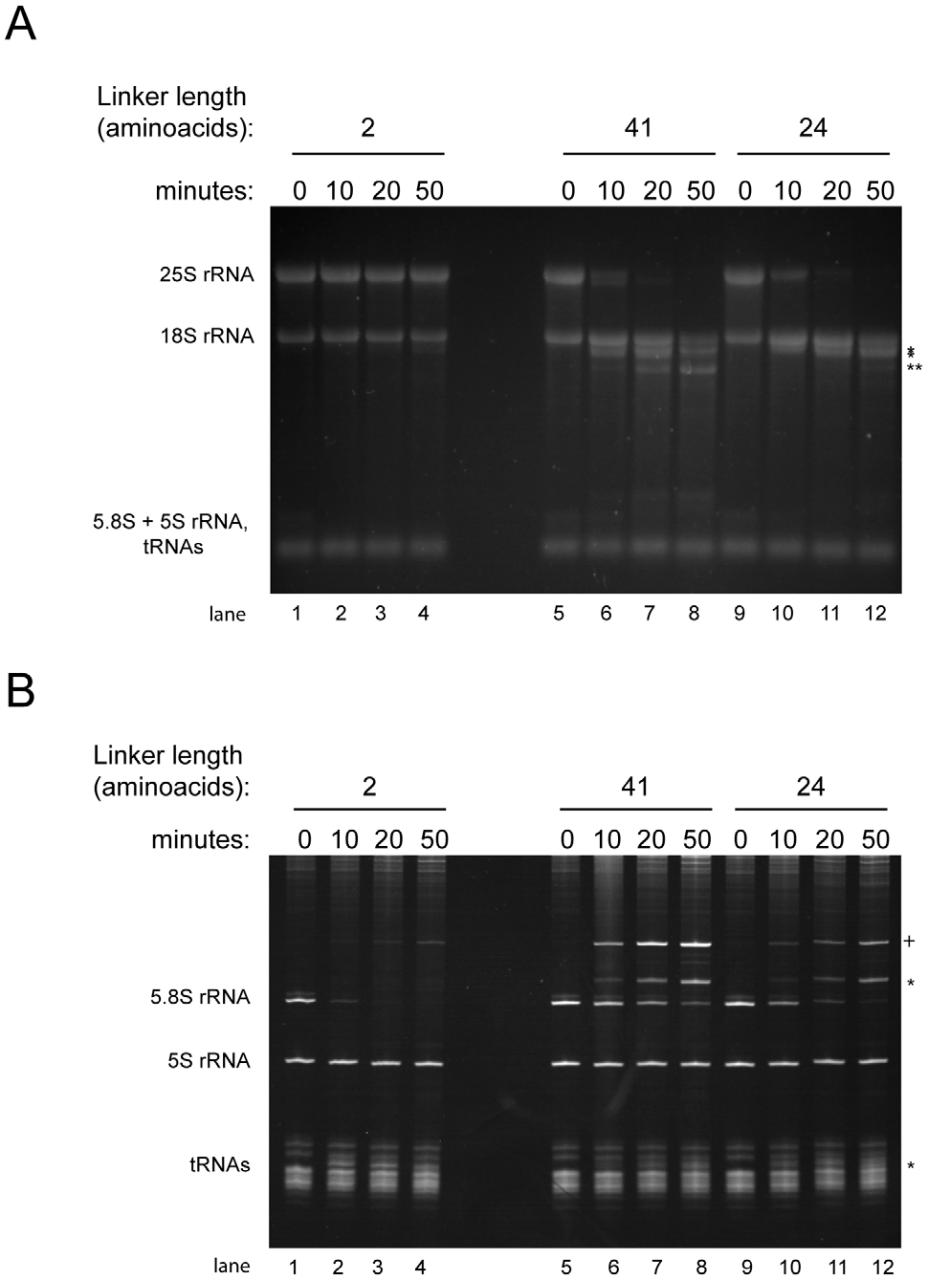
Influence of the linker size of MNase-rpL35 fusion proteins on rRNA fragmentation. Cellular extracts of yeast strains expressing MNAse fusion proteins of rpL35 in which the MNase and the rpL35 coding regions are separated by 2 (Y2510, lanes 1–4), 41 (Y2511, lanes 5–8) or 24 (Y2512, lanes 9–12) amino acids were prepared as described in Materials and Methods. Samples were taken before addition of calcium chloride (0 minutes) or after incubation in the presence of calcium chloride for the indicated times at room temperature (22°C). Total RNA was extracted and separated by size on agarose gels (A) or on polyalcrylamide gels (B) as described in Materials and Methods. Gels were stained with ethidium bromide. Positions of 25S rRNA, 18S rRNA, 5.8S rRNA, 5S rRNA and tRNAs are indicated. Stars (*) indicate positions of strain specific rRNA fragments generated during the course of extract incubation in the presence of calcium chloride. Two stars (**) indicate positions of rRNA fragments appearing in higher amounts in the strain expressing MNAse linked to rpL35 by 41 amino acids. A cross (+) marks a RNA fragment significantly detected during the time course of incubation in strains not expressing MNase fusion proteins.

Inspection of atomic resolution three dimensional structure models of yeast ribosomes [11] revealed that the major cuts of MNase-rpL5 were localized in the central protrusion next to the rpL5 binding site (Figs. 4A and 4B). Cuts of MNase-rpL35 were localized on the opposite side of the ribosome clustered around the rpL35 binding site around the exit tunnel (Figs. 4A and 4C). Measurements on the basis of the atomic coordinates provided in [11] indicated that the two major MNase-rpL5 cuts were about 1.5 and 4 nm, respectively, away from the rpL5 amino terminus. The MNase-rpL35 proximal cut in 5.8S rRNA was about 1 nm away from the rpL35 amino terminus while the two distal cuts in the 25S rRNA were between 5 and 5.5 nm away. We reasoned that the nucleolytic actions of MNAse fusion proteins detected in these experiments were defined by a few major determinants: 1) The MNase substrate preference for single stranded, non-protein covered pyrimidines [7]. 2) The local tethering of MNase through the ribosomal protein fusions. 3) The conformational flexibility of the surrounding rRNA. And 4) the (flexibility in) distance between the MNase active center and the fused ribosomal proteins. MNase is a relatively small (19 kD) globular protein and the distance between the MNases active center and the first carboxyterminal amino acid seen in X-ray structure analyses is about 2.5 nm (pdb file 1SNC, [16]). In case of the fusion proteins used here, the MNase active domain was separated from ribosomal proteins by a few additional amino acids from the unstructured MNase C-terminus together with 24 vector backbone encoded amino acids coding for two HA epitope tags. To test whether the size of the linker separating MNase from ribosomal proteins has an impact on the observed fragmentation patterns, we constructed plasmids allowing the expression of MNase-rpL35 fusion proteins with longer (41 amino acids) or shorter (2 amino acids) linker sequences. The 41 amino acids containing linker consisted of two HA epitope tags followed by a serine and glycine rich sequence (GGGGSGGGSGGGSGGGS) predicted to fold as a random coil [17], [18]. Genetic analyses indicated that the new constructs complemented for the essential functions of RPL35 (data not shown). Clear differences in the rRNA fragmentation pattern in calcium treated extracts from the MNase-rpL35 expressing strains were observed (Fig. 5). As shown before (Figs. 1A and 2A), in ethidium bromide stained gels a specific fragmentation pattern of 25S rRNA and 5.8S rRNA was detected in extracts containing MNase fused to rpL35 through a 24 amino acids linker (Figs. 5A and 5B, lanes 9–12). In case of a 41 amino acids linker, 5.8S rRNA degradation was slightly delayed (Fig. 5B, compare 5.8S rRNA signals in lanes 5–8 and 9–12) and appearance of some additional rRNA fragments running slightly lower than 18S rRNA was evident (Fig. 5A lanes 7 and 8, marked by two stars). The observed delay in cleavage at the rpL35 proximal site in 5.8S rRNA might be due to the fold of the larger linker favoring a location of the MNase portion more distal to rpL35. For extracts containing MNase fused through a 2 amino acids linker to rpL35, fragmentation of 25S rRNA was slowed down (Fig. 5A, compare 25S rRNA signals in lanes 1–4 and lanes 9–12), while the MNase-rpL35 proximal cut in the 5.8S rRNA occurred with significantly faster kinetics (Fig. 5B, compare 5.8S rRNA signal in lanes 1–4 with lanes 9–12). Thus, a shorter linker favored cleavages close to rpL35, and larger linkers favored cleavages more distal to rpL35. These observations indicated that careful design of the linker between MNase and the RNP component allow to influence the radius in which RNA elements surrounding the fusion proteins in an RNP can be probed.

## Discussion

The data presented here indicate that fusion proteins of MNase with RNA interacting proteins can be useful tools for local structure probing within RNPs. It is relatively easy to apply using standard methods of molecular biology and the RNP to be studied can be fully assembled *in vivo*. We think that the approach is specifically attractive to study the spatial interrelationship between proteins and RNA elements in RNPs which show a certain amount of conformational flexibility or contain some regions with a high degree of conformational flexibility hardly addressable by techniques like X-ray crystallography. It could add valuable information on the three dimensional organization of an RNP when combined with global secondary structure analyses [2] and when the RNA binding sites of the protein used for probing were already identified by UV cross linking techniques [19], [20]. An appealing option is to influence the local space to be analyzed in a given RNP by changing the length and characteristics of the linker sequences connecting the MNase with the fusion proteins. The linkers tested in this work were all predicted to fold as random coil and to allow a rather high degree of conformational flexibilities. Choice of less flexible, helix forming linkers [17] might allow to specifically probe for more distant MNase sensitive RNA elements.

Obvious disadvantages for certain applications include the intrinsic substrate specificity of MNase. Directed hydroxyl radical probing [4], an alternative approach, guarantees a substantially higher resolution. Here, hydroxyl radicals are generated at a specific site in the RNP via tethering of iron (II) ions to single cysteins in the recombinant protein used for probing. High amounts of RNA backbone cleavages are observed in this case in a radius of up to 4.4 nm surrounding the *in vitro* modified cystein residue. We would predict however for 3D structure probing with MNase that the number of MNase accessible sites, and thereby the resolution, can increase for RNPs with a less rigid and compact fold as mature ribosomes. It seems moreover an option to consider nucleases with other substrate specificities as alternative fusion proteins or to screen for MNase variants with different substrate preferences.

Further improvement of the approach described here could come from using methods like RNA sequencing instead of primer extension analyses and Northern blotting to analyze the occurring nucleolytic events in a less biased and more sensitive way. As shown by Laemmli and co-workers, another attractive possibility is to activate MNase fusion proteins already *in vivo* through permeabilization of cells with digitonin followed by the addition of calcium [9]. Finally, we consider that the approach described here could find further applications, as in the targeted fragmentation of RNPs. Local cleavages in some regions of RNA could be produced by MNase fusion proteins and the effects on structural integrity and residual association of other protein components could be analyzed. Another application might be the *de novo* identification of RNA constituents of heterogenous RNPs. Accordingly, a common protein component of heterogeneous cellular RNPs could be expressed in fusion with MNase. The fusion protein selectively degrades RNA in proximity to the fusion protein after activation of the MNase through addition of calcium to cellular extracts. Degraded RNA could then be identified by comparing the RNA composition of extracts treated or not with calcium.

## Materials and Methods

### Yeast Methods and Plasmid Construction

For a complete list of the oligonucleotides and plasmids used in this work and of their design see Figs. S1 and S2. Fig. S3 shows a list of the yeast strains used in this work including a description of their genotypes. Transformation of yeast cells and their cultivation in full or synthetic medium were done according to standard protocols [21], [22].

### MNase Activation in Cellular Extracts

Cells from 100 ml of yeast culture grown in full medium (YPD) to an optical density of 1 (OD600) were harvested by centrifugation at room temperature (3000 g) and were washed once in 20 ml ice cold water and twice in 1 ml ice cold buffer AG200 (200 mM potassium chloride, 20 mM Tris pH8, 5 mM magnesium acetate, 5 mM EGTA pH8, 1 mM PMSF, 2 mM Benzamidine). 1.5 ml of cold buffer AG200 per gram of wet cell pellet were added and the resulting suspension was shaken in the presence of glass beads (1.4 g of 1 mm diameter glass beads per 0.8 ml suspension in a tube of 2 ml volume) on a vibrax mixer (IKA) at 4°C and maximum speed three times for 5 minutes. The extract was clarified twice by centrifugation for 5 and 10 minutes at 13000 g and 4°C. Protein concentrations of the clarified extracts were determined by Bradford protein assay (Bio-Rad Laboratories). MNase was activated by incubation of the extracts at 22°C in the presence of 8 mM calcium chloride. To stop the reaction, extract volumes corresponding to 200 µg of proteins were added to 500 µl ice cold buffer AE+ (50 mM sodium acetate pH 5.3, 20 mM EDTA) and samples were frozen at −20°C. Subsequently, RNA extraction by hot acidic phenol treatment was performed as described in [23].

### Northern Blotting and Primer Extension Analyses

RNA separation on formaldehyde/MOPS agarose gels or urea/TBE/polyacrylamide gels and subsequent Northern blotting analyses were done essentially as described in [24]. Hybridization with probes was performed in 50% formamide, 5× SSC, 0,5% SDS, 5× Denhardt’s solution at 30°C with the indicated ^32^P-labelled probes (See Fig. S1 for a list of oligonucleotides used in this study). The blots were washed twice for 15 min with 2x SSC at 30°C. Signals were detected using a PhosphorImager FLA3000 (Fujifilm) and data were quantified using MultiGauge V3.0 (Fujifilm).

Primer extension analyses were done as described in [25] using primers indicated in the figures legends. Sequencing reactions using the same primers were performed with the Thermo Sequenase cycle sequencing kit (785001KT Affymetrix). Products of sequencing reactions and of primer extension analyses were size separated on urea/taurin/polyacrylamide gels according to the manufacturers instructions.

### Affinity Purification of MNAse Fusion Proteins

Total cellular extracts of cells from 500 ml culture were prepared as described above. A volume of extract containing about 10 mg of total protein was adjusted to 0.5% Triton-X100 and 0.1% Tween 20 and loaded onto 50 µl of anti HA affinity matrix (Roche Diagnostics) equilibrated in buffer AG200 containing 0.5% Triton X-100 and 0.1% Tween20. After one hour incubation at 4°C the matrix was transferred into a poly prep chromatography column (Bio-Rad Laboratories) and washed three times with 2 ml and one time with 10 ml AG200 containing 0.5% Triton-X100 and 0.1% Tween 20. After an additional wash step with 1 ml of buffer AG200 the affinity matrix was suspended in 1 ml AG200 and split into two parts which were centrifuged for 2 minutes at 2000 g and 4°C to discard the supernatant. One half of the affinity matrix was suspended in SDS sample buffer and further analyzed by SDS polyacrylamide gel electrophoresis (16% acrylamide gels were supplemented with 4.5 M Urea) and Coomassie staining or Western blotting. Total RNA was extracted from the other half and analyzed by agarose or polyacrylamide/TBE gel electrophoresis followed by ethidium bromide staining.

## Supporting Information

Figure S1 **Oligonucleotides used in this study.**
(PDF)

Figure S2 **Plasmids used in this study.**
(PDF)

Figure S3 **Yeast strains used in this study.**
(PDF)

Figure S4 **Possible mode of action of a nuclease fusion protein incorporated into an RNP.** The figure legend in the lower panel gives a description of the symbols used to represent specific structural features of the RNP and the nuclease fusion protein. The left and right part of the figure show two different conformational states of the RNP which might be the consequence of changes in the RNP’s interaction partners. Small arrows and transparent shapes of the linker, the nuclease and nuclease sensitive sites indicate varying local conformations. In the linear RNA sequence the RNA binding sites of the tested protein can be far away from the respective RNA cleavages made by the fused nuclease.
(PDF)

Figure S5 **Growth curves of yeast strains BY4741, Y2361, Y2369 and Y2371.** Yeast strains Y206 (wildtype BY4741), Y2361 (MNase-rpS13), Y2369 (MNase-rpL5) and Y2371 (MNase-rpL35) were grown overnight in YPD medium and then diluted to an OD_600_ of 0.02 in 0.2 ml of fresh YPD in a covered 96 well plate. Cells were incubated at 30°C in a TECAN infinite F500 reader with measurements taken in kinetic cycle mode (shaking for a duration of 25 seconds per cycle in orbital shaking mode with an amplitude of 5 mm, wait time of 30 seconds before measurement of the OD_612_, total cycle length of 15 minutes). Generation times in logarithmic growth phase of strains Y2369 (MNase-rpL5) and Y2371 (MNase-rpL35) were increased less than 15% compared to the one of the wildtype strain Y206 (103 and 104 minutes versus 93 minutes). Generation time of Y2361 (MNase-rpS13) was increased less than 40% (128 minutes versus 93 minutes). Growth measurements using larger culture volumes incubated at 30°C in Erlenmeyer flasks on a rotary shaker gave identical results in regard to these relative changes in generation times.
(PDF)

Figure S6 **MNase fusion proteins of rpS13, rpL5 and rpL35 expressed in yeast strains Y2361, Y2369 and Y2371 get incorporated into ribosomal particles.** Cellular extracts of yeast strains which express no MNase fusion protein (Y206, wildtype BY4741, lanes 1 and 5) or fusions of MNase linked with rpS13 (Y2361, lanes 2 and 6), rpL5 (Y2369, lanes 3 and 7) or rpL35 (Y2371, lanes 4 and 8) by two consecutive HA tags were used for affinity purification with an anti-HA affinity matrix as described in materials and methods. Protein composition of total cellular extracts (lanes 1–4) and affinity purified fractions (lanes 5–8) were further analyzed by SDS PAGE analyses and Coomassie staining (upper panel) and Western blotting (upper middle panel, anti HA antibody 3F10) as described in materials and methods. Migration behaviour of marker proteins with the indicated molecular weight is depicted on the left. Coomassie bands marked with a star were specifically detected in affinity purified fractions of MNAse-2xHA-rpS13, MNase-2xHA-rpL5 or MNase-2xHA-rpL35 and their migration behaviour in SDS PAGE analyses was consistent with the expected molecular weight of these fusion proteins. RNA composition of total cellular extracts (lanes 1–4) and affinity purified fractions was further analyzed by agarose gel electrophoresis (lower middle panel) or polyacrylamide gel electrophoresis (lower panel) followed by ethidium bromide staining. Positions of 25S rRNA, 18S rRNA, 5.8S rRNA, 5S rRNA and tRNAs are indicated. Volume percent of fractions used for the respective analyses are indicated on the right in brackets. We note the slightly increased 5S rRNA to 5.8S rRNA ratio in affinity purified fractions of MNase-2xHA fusions with rpL5 (lane 7), which is a component of the 5S rRNP.
(PDF)

Figure S7 **Major cleavage events in extracts of strain Y2371 depend on the addition of exogenous calcium ions.** A cellular extract of yeast strain Y2371 expressing rpL35 in fusion with MNAse was prepared as described in Materials and Methods. Calcium chloride was added to one part of the extract (+, lanes 1 and 3) and omitted from the other part (−, lanes 2 and 4). Samples were taken before (0 minutes) or after extract incubation for 40 minutes at room temperature (22°C). Total RNA was extracted and separated by size on denaturing polyacrylamide gels or on agarose gels as described in Materials and Methods. Gels were stained with ethidium bromide. Positions of 25S rRNA, 18S rRNA 5.8S rRNA, 5S rRNA and tRNAs are indicated. Stars (*) indicate positions of strain specific rRNA fragments generated during the course of extract incubation in the presence of calcium chloride (see Figs. 1 and 2).
(PDF)

Figure S8 **rRNA degradation in yeast cellular extracts after addition of exogenous MNase.** Cellular extracts of yeast strain Y206 (wildtype BY4741) were prepared in buffer AG200 as described in materials and methods. Purified recombinant MNase was added in a concentration of 0.5U per µg yeast protein (lanes 1–5) or in a concentration of 0.2U per µg yeast protein (lanes 6–10). Calcium chloride was added to a final concentration of 7 mM and the extracts were incubated at room temperature (22°C). Samples were taken after 0 minutes (lanes 1 and 6), after 5 minutes (lanes 2 and 7), after 10 minutes (lanes 3 and 8), after 20 minutes (lanes 4 and 9) and after 40 minutes (lanes 5 and 10) of incubation times. Total RNA was extracted and analyzed by agarose gel electrophoresis (upper panel) or by denaturing TBE/polyacrylamide gel electrophoresis (lower panel) followed by ethidium bromide staining as described in materials and methods. 25S rRNA, 18S rRNA, 5.8S rRNA, 5S rRNA and tRNA visible in lanes 1 and 6 are labeled on the left.
(PDF)

Figure S9 **Map of 25S rRNA and 5.8S rRNA including positions of oligonucleotides used in this study and the major cuts observed in yeast strains Y2371 and Y2369.** Oligonucleotides are represented by red boxes, major cleavage events by blue arrows and resulting rRNA fragments by grey lines.
(PDF)
