# Supplementary material for: Local Tertiary Structure Probing of Ribonucleoprotein Particles by Nuclease Fusion Proteins
Source: PLoS One. 2012 Aug 2;7(8):e42449. doi: 10.1371/journal.pone.0042449 (PMC3411627; doi:10.1371/journal.pone.0042449)
Supplement: Figure S1 — Oligonucleotides used in this study. (PDF) [file pone.0042449.s001.pdf]

Supplementary Figure S1. Oligonucleotides used in this study

Number	Name	Sequence (5‘ to 3‘ direction)
O209	o6-5.8	TTTCGCTGCGTTCTTCATC
O1821	C1+70bp	TGCCGCTTCACTCGCCGTTAC
O1890	25S-DomIII_rev	CAACTAGAGGCTGTTCACCTTGGA
O1896	25S-DomVI_rev	ACAAATCAGACAACAAAGGCTTAA
O1957	18S-DomIII_rev	TAATGATCCTTCCGCAGGTTCACC
O2474	5S-rDNA	TTAACTACAGTTGATCGG
O2959	5.8SrRNA 3´ probe	AAATGACGCTCAAACAGGCAT
O3068	25S+1645	TTACGTTACCGTGAAGAATCC
O3074	25S+1088	ACGTTCAATTAAGTAACAAGG
O464	RPS13E GAL F	CGCCGCGGATCCATGGGTCGTATGCACAGTGCCGG TAAAGGTATTTCTTCTTCTGCTATT
O466	RPS5 GAL F	CGCCGCGGATCCATGTCTGACACCGAAGCT
O581	RPS28B-Prom-F2	TTTTTTGAATTCGCTTATTCATGTTCGAATC
O903	RPS5_rev_PstI	GCGCTGCAGGTAGAGTGACTTAGAAA
O947	Yc-KpnI-Up	GATCTGGTACCGGATCCTCTAGAGTCGACCTGCA
O948	Yc-KpnI-Do	GGTCGACTCTAGAGGATCCGGTACCA
O949	pRPS28B-Rev-BamHI	TTTTTTGGATCCTGCTGCTCTTTTATGCTTTGC
O981	RPS13-Term-Pst	TTTTTTCTGCAGAACGGTCCGATATAAGCT
O2911	MnaseCloning_Re	CCTGAGAAAGCAACCTGACC
O2912	MnaseCloning_Fo	TTTTTTGGTACCATGAACGCAACTTCAACTAAAAAATT AC
O3081	MNase-vorStop-Rev	TTTTTTGGATCCACCTGAATCAGCGTTGTCTTCG
O3082	MNAse-ExtLink-Rev	TTTTTTGGATCCAGAGCCTCCCCCAGATCCGCCACCT GATCCACCGCCGCTACCGCCTCCTCCTGCATAGTCC GGGACGTC
