# Supplementary material for: Local Tertiary Structure Probing of Ribonucleoprotein Particles by Nuclease Fusion Proteins
Source: PLoS One. 2012 Aug 2;7(8):e42449. doi: 10.1371/journal.pone.0042449 (PMC3411627; doi:10.1371/journal.pone.0042449)
Supplement: Figure S2 — Plasmids used in this study. (PDF) [file pone.0042449.s002.pdf]

Supplementary Figure S2. Plasmids used in this study

Number	Name	Yeast Marker	Origin/Construction
K230	Ycplac111-pGAL	LEU2	Ferreira-Cerca et al. 2005 [1]
K375	pT11		contains the rDNA locus of S. cerevisiae, more details are available upon request
K643	pKM9		Merz et al. 2008 [2]
K615	pGAL-RPS13	LEU2	PCR product (oligos O464 and O981 on yeast genomic DNA) was cloned with BamHI/PstI into K230
K852	pGAL-RPL5	LEU2	Pöll et al. 2009 [3]
K857	pGAL-RPL35A	LEU2	Pöll et al. 2009 [3]
K1735	Yep-pRPS28Mnase-RPS5	URA3	The vector K1735 is based on vector Yeplac195 [4] into which a PCR amplicon (oligos O581 and O949, yeast genomic DNA as template) containing the promoter of RPS28B was cloned with EcoRI and BamHI. A KpnI site was introduced upstream of the BamHI site by cloning a hybrid of oligos O947 and O948 between the BamHI and PstI sites. A PCR amplicon (oligos O2911 and O2912 and vector K643 as template) containing the MNase coding region followed by two HA epitope coding regions was cloned between the KpnI and BamHI sites. The vector contains in addition to that the yeast RPS5 coding region together with the following 300 3' untranslated nukleotides (PCR with oligos O466 and O903 on yeast genomic DNA) cloned between the BamHI and PstI sites and a Flag tag coding region between the PstI and HindIII sites.
K1738	Yep-pRPS28Mnase-RPS13	URA3	A BamHI-PstI Fragment containing the RPS13 coding region was cloned from K615 into K1735
K1742	Yep-pRPS28Mnase-RPL5	URA3	A BamHI-PstI Fragment containing the RPL5 coding region was cloned from K852 into K1735
K1743	Yep-pRPS28Mnase-RPL35	URA3	A BamHI-PstI Fragment containing the RPL35A coding region was cloned from K857 into K1735
K1940	Yep-pRPS28Mnase-Slink	URA3	The vector K1940 is based on vector Yeplac195 [4] into which a PCR amplicon (oligos O581 and O949, yeast genomic DNA as template) containing the promoter of RPS28B was cloned with EcoRI and BamHI. A KpnI site was introduced upstream of the BamHI site by cloning a hybrid of oligos O947 and O948 between the BamHI and PstI sites. A PCR amplicon (oligos O3081 and O2912 and vector K643 as template) containing the MNase coding region was cloned between the KpnI and BamHI sites. The vector contains in addition to that a Flag tag coding region between the PstI and HindIII sites
K1941	Yep-pRPS28Mnase-Llink	URA3	The vector K1941 is based on vector

			Yeplac195 [4] into which a PCR amplicon (oligos O581 and O 949, yeast genomic DNA as template) containing the promoter of RPS28B was cloned with EcoRI and BamHI. A KpnI site was introduced upstream of the BamHI site by cloning a hybrid of oligos O947 and O948 between the BamHI and PstI sites. A PCR amplicon (oligos O3082 and O2912 and vector K643 as template) containing the MNase coding region followed by two HA epitope coding regions and an additional linker region was cloned between the KpnI and BamHI sites. The vector contains in addition to that a Flag tag coding region between the PstI and HindIII sites.
K1943	pRPS28-Mnase-Slink-L35	URA3	A BamHI/PstI Fragment containing the RPL35A coding region was cloned from K857 into K1940
K1946	pRPS28-Mnase-Llink-L35	URA3	A BamHI/PstI Fragment containing the RPL35A coding region was cloned from K857 into K1941

1. Ferreira-Cerca S, Pöll G, Gleizes P-E, Tschochner H, Milkereit P (2005) Roles of eukaryotic ribosomal proteins in maturation and transport of pre-18S rRNA and ribosome function. Mol Cell 20: 263–275. doi:10.1016/j.molcel.2005.09.005.

2. Merz K, Hondele M, Goetze H, Gmelch K, Stoeckl U, et al. (2008) Actively transcribed rRNA genes in S. cerevisiae are organized in a specialized chromatin associated with the high-mobility group protein Hmo1 and are largely devoid of histone molecules. Genes Dev 22: 1190–1204. doi:10.1101/gad.466908.

3. Pöll G, Braun T, Jakovljevic J, Neueder A, Jakob S, et al. (2009) rRNA maturation in yeast cells depleted of large ribosomal subunit proteins. PLoS ONE 4: e8249. doi:10.1371/journal.pone.0008249.

4. Gietz RD, Sugino A (1988) New yeast-Escherichia coli shuttle vectors constructed with in vitro mutagenized yeast genes lacking six-base pair restriction sites. Gene 74: 527–534.
