# Supplementary material for: Local Tertiary Structure Probing of Ribonucleoprotein Particles by Nuclease Fusion Proteins
Source: PLoS One. 2012 Aug 2;7(8):e42449. doi: 10.1371/journal.pone.0042449 (PMC3411627; doi:10.1371/journal.pone.0042449)
Supplement: Figure S3 — Yeast strains used in this study. (PDF) [file pone.0042449.s003.pdf]

Supplementary Figure S3. Yeast strains used in this study

number	Name	Plasmids	Genotype	Origin
Y206	BY4741		his31<br>leu20<br>met150<br>ura30	Euroscarf
Y701	pGAL-RPS13	K615 (LEU2/CEN, pGAL-RPS13)	his31<br>leu20<br>ura30<br>MET15<br>LYS2<br>YDR064w::kanMX4	Ferreira-Cerca et al., 2005 [1]
Y1092	pGAL-RPL5	K852 (LEU2/CEN, pGAL-RPL5)	his3-1<br>leu2-0<br>ura3-0<br>YPL131w::kanMX4	Pöll et al., 2009 [2]
Y1094	pGAL-RPL35	K857 (LEU2/CEN, pGAL-RPL35)	his3-1<br>leu2-0<br>ura3-0<br>YDL191w::kanMX4<br>YDL136w::HIS3	Pöll et al., 2009 [2]
Y2361	pRPS28-Mnase-RPS13	K1738 (URA3/2u, pRPS28-MNase-rpS13)	his31<br>leu20<br>ura30<br>YDR064w::kanMX4	Strain Y701 was transformed with K1738, selection on YNGal-URA, then growth on YPD, minor growth phenotype was detected
Y2369	pRPS28-Mnase-RPL5	K1742(URA3/2u, pRPS28-MNase-rpL5)	his3-1<br>leu2-0<br>ura3-0<br>YPL131w::kanMX4	Strain Y1092 was transformed with K1742, selection on YNGal-URA, then growth on YPD
Y2371	pRPS28-Mnase-RPL35	K1743 (URA3/2u, pRPS28-MNase-rpL35)	his3-1<br>leu2-0<br>ura3-0<br>YDL191w::kanMX4<br>YDL136w::HIS3	Strain Y1094 was transformed with K1743 , selection on YNGal-URA, then growth on YPD
Y2510	pRPS28-MNase-SLink-RPL35	K1943 (URA3/2u, pRPS28-MNase-SLink-rpL35)	his3-1<br>leu2-0<br>ura3-0<br>YDL191w::kanMX4<br>YDL136w::HIS3	Strain Y1094 was transformed with K1943, selection on YNGal-URA, then growth on YPD
Y2511	pRPS28-MNase-LLink-RPL35	K1946 (URA3/2u, pRPS28-MNase-LLink-rpL35)	his3-1<br>leu2-0<br>ura3-0<br>YDL191w::kanMX4<br>YDL136w::HIS3	Strain Y1094 was transformed with K1946, selection on YNGal-URA, then growth on YPD
Y2512	pRPS28MNase-MLink-RPL35	K1743 (URA3/2u, pRPS28-MNase-MLink-rpL35)	his3-1<br>leu2-0<br>ura3-0<br>YDL191w::kanMX4<br>YDL136w::HIS3	Strain Y1094 was transformed with K1743 selection on YNGal-URA, then growth on YPD

1. Ferreira-Cerca S, Pöll G, Gleizes P-E, Tschochner H, Milkereit P (2005) Roles of eukaryotic ribosomal proteins in maturation and transport of pre-18S rRNA and ribosome function. Mol Cell 20: 263–275. doi:10.1016/j.molcel.2005.09.005.

2. Pöll G, Braun T, Jakovljevic J, Neueder A, Jakob S, et al. (2009) rRNA maturation in yeast cells depleted of large ribosomal subunit proteins. PLoS ONE 4: e8249. doi:10.1371/journal.pone.0008249.
