# Supplementary figures and images for: Local Tertiary Structure Probing of Ribonucleoprotein Particles by Nuclease Fusion Proteins
Source: PLoS One. 2012 Aug 2;7(8):e42449. doi: 10.1371/journal.pone.0042449 (PMC3411627; doi:10.1371/journal.pone.0042449)

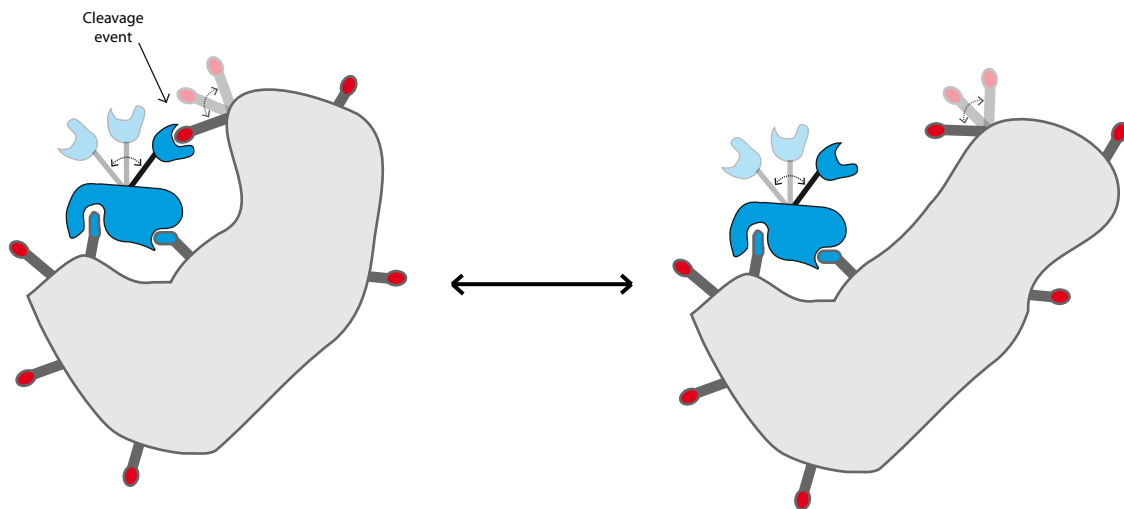

### Legend:

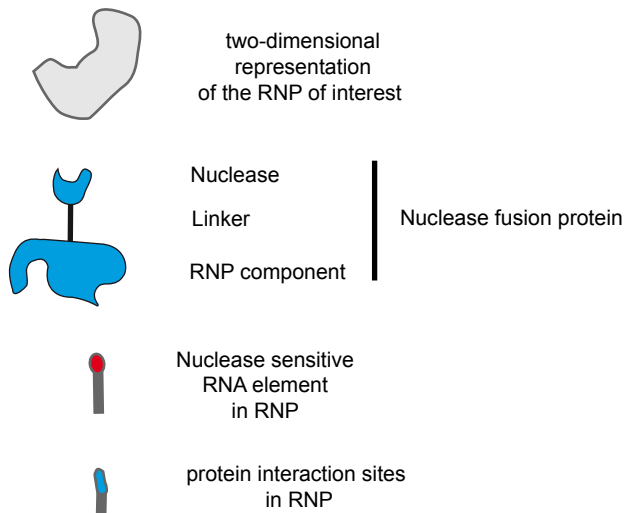

Supplement: Figure S4 — Possible mode of action of a nuclease fusion protein incorporated into an RNP. The figure legend in the lower panel gives a description of the symbols used to represent specific structural features of the RNP and the nuclease fusion protein. The left and right part of the figure show two different conformational states of the RNP which might be the consequence of changes in the RNP’s interaction partners. Small arrows and transparent shapes of the linker, the nuclease and nuclease sensitive sites indicate varying local conformations. In the linear RNA sequence the RNA binding sites of the tested protein can be far away from the respective RNA cleavages made by the fused nuclease. (PDF) [file pone.0042449.s004.pdf]

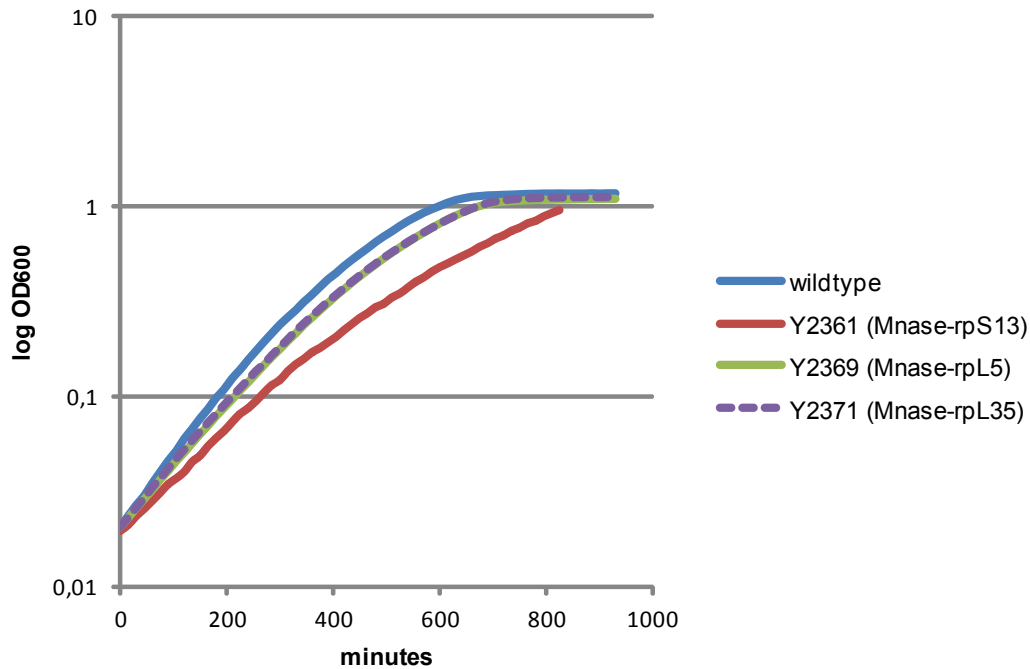

Supplement: Figure S5 — Growth curves of yeast strains BY4741, Y2361, Y2369 and Y2371. Yeast strains Y206 (wildtype BY4741), Y2361 (MNase-rpS13), Y2369 (MNase-rpL5) and Y2371 (MNase-rpL35) were grown overnight in YPD medium and then diluted to an OD600 of 0.02 in 0.2 ml of fresh YPD in a covered 96 well plate. Cells were incubated at 30°C in a TECAN infinite F500 reader with measurements taken in kinetic cycle mode (shaking for a duration of 25 seconds per cycle in orbital shaking mode with an amplitude of 5 mm, wait time of 30 seconds before measurement of the OD612, total cycle length of 15 minutes). Generation times in logarithmic growth phase of strains Y2369 (MNase-rpL5) and Y2371 (MNase-rpL35) were increased less than 15% compared to the one of the wildtype strain Y206 (103 and 104 minutes versus 93 minutes). Generation time of Y2361 (MNase-rpS13) was increased less than 40% (128 minutes versus 93 minutes). Growth measurements using larger culture volumes incubated at 30°C in Erlenmeyer flasks on a rotary shaker gave identical results in regard to these relative changes in generation times. (PDF) [file pone.0042449.s005.pdf]

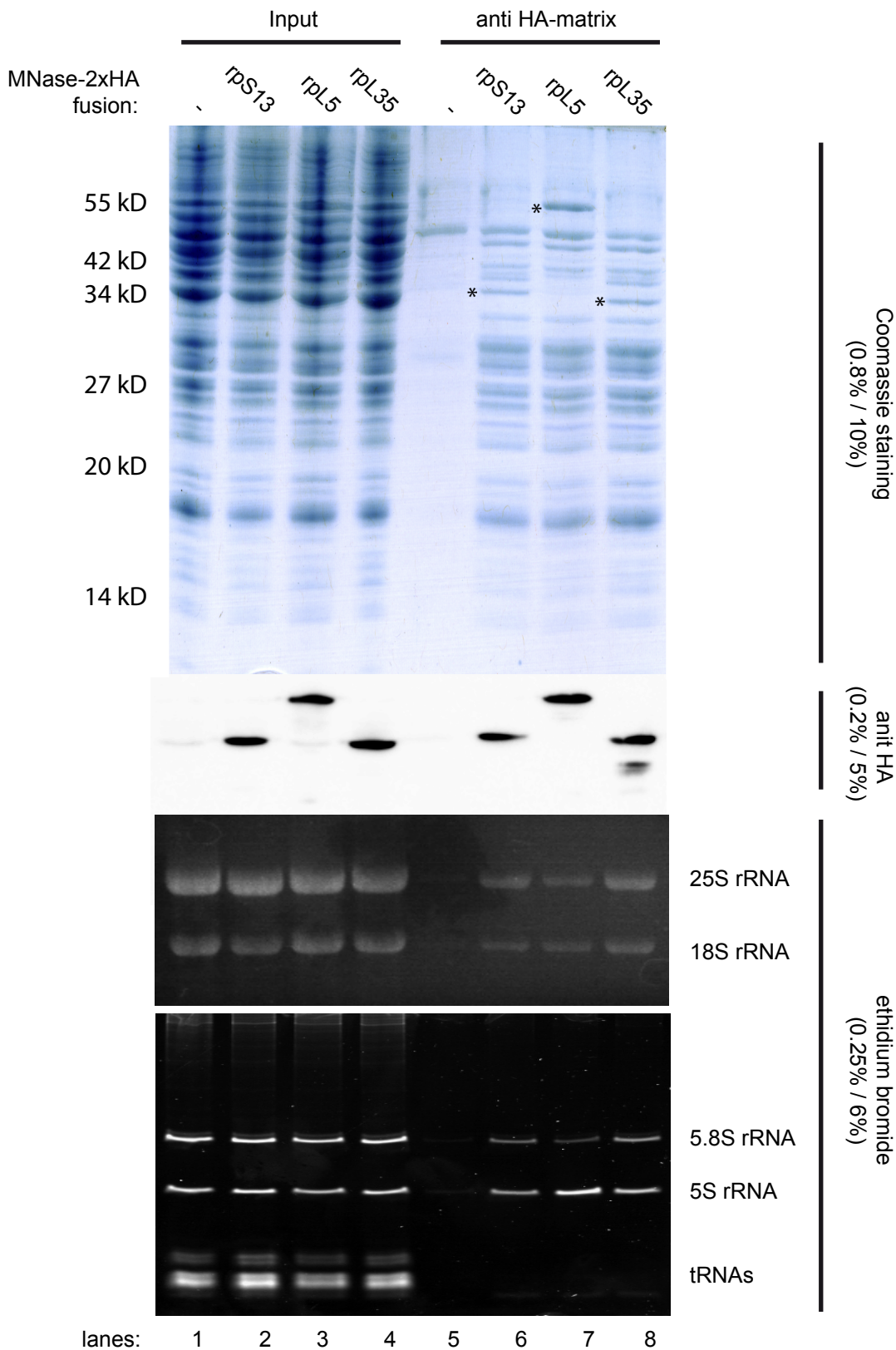

Supplement: Figure S6 — MNase fusion proteins of rpS13, rpL5 and rpL35 expressed in yeast strains Y2361, Y2369 and Y2371 get incorporated into ribosomal particles. Cellular extracts of yeast strains which express no MNase fusion protein (Y206, wildtype BY4741, lanes 1 and 5) or fusions of MNase linked with rpS13 (Y2361, lanes 2 and 6), rpL5 (Y2369, lanes 3 and 7) or rpL35 (Y2371, lanes 4 and 8) by two consecutive HA tags were used for affinity purification with an anti-HA affinity matrix as described in materials and methods. Protein composition of total cellular extracts (lanes 1–4) and affinity purified fractions (lanes 5–8) were further analyzed by SDS PAGE analyses and Coomassie staining (upper panel) and Western blotting (upper middle panel, anti HA antibody 3F10) as described in materials and methods. Migration behaviour of marker proteins with the indicated molecular weight is depicted on the left. Coomassie bands marked with a star were specifically detected in affinity purified fractions of MNAse-2xHA-rpS13, MNase-2xHA-rpL5 or MNase-2xHA-rpL35 and their migration behaviour in SDS PAGE analyses was consistent with the expected molecular weight of these fusion proteins. RNA composition of total cellular extracts (lanes 1–4) and affinity purified fractions was further analyzed by agarose gel electrophoresis (lower middle panel) or polyacrylamide gel electrophoresis (lower panel) followed by ethidium bromide staining. Positions of 25S rRNA, 18S rRNA, 5.8S rRNA, 5S rRNA and tRNAs are indicated. Volume percent of fractions used for the respective analyses are indicated on the right in brackets. We note the slightly increased 5S rRNA to 5.8S rRNA ratio in affinity purified fractions of MNase-2xHA fusions with rpL5 (lane 7), which is a component of the 5S rRNP. (PDF) [file pone.0042449.s006.pdf]

|          |   |   |    |    |
|----------|---|---|----|----|
| minutes: | 0 | 0 | 40 | 40 |
| Ca++:    | + | - | +  | -  |

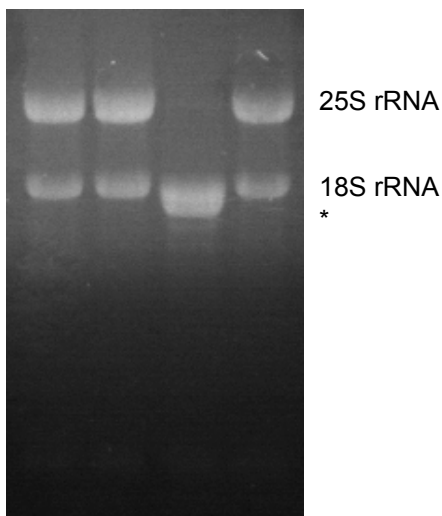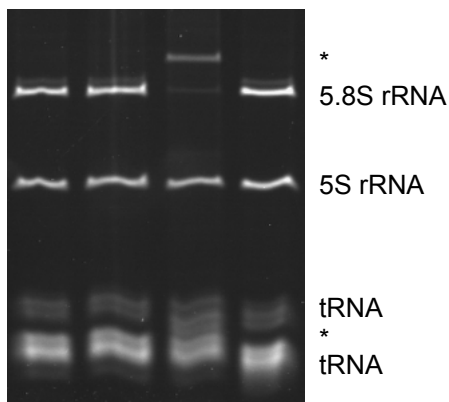

|        |   |   |   |   |
|--------|---|---|---|---|
| lanes: | 1 | 2 | 3 | 4 |
|--------|---|---|---|---|

Supplement: Figure S7 — Major cleavage events in extracts of strain Y2371 depend on the addition of exogenous calcium ions. A cellular extract of yeast strain Y2371 expressing rpL35 in fusion with MNAse was prepared as described in Materials and Methods. Calcium chloride was added to one part of the extract (+, lanes 1 and 3) and omitted from the other part (−, lanes 2 and 4). Samples were taken before (0 minutes) or after extract incubation for 40 minutes at room temperature (22°C). Total RNA was extracted and separated by size on denaturing polyacrylamide gels or on agarose gels as described in Materials and Methods. Gels were stained with ethidium bromide. Positions of 25S rRNA, 18S rRNA 5.8S rRNA, 5S rRNA and tRNAs are indicated. Stars (*) indicate positions of strain specific rRNA fragments generated during the course of extract incubation in the presence of calcium chloride (see Figs. 1 and 2). (PDF) [file pone.0042449.s007.pdf]

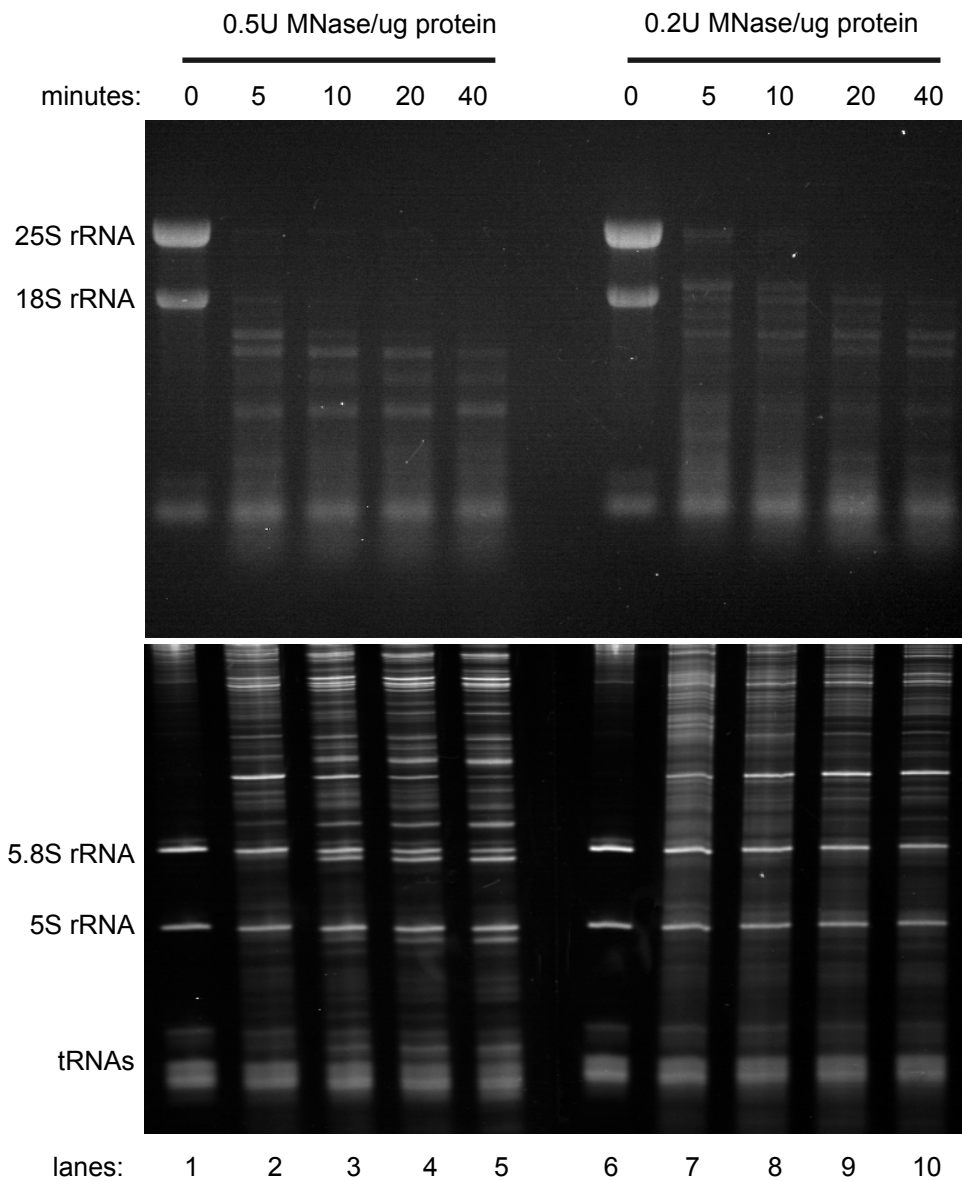

Supplement: Figure S8 — rRNA degradation in yeast cellular extracts after addition of exogenous MNase. Cellular extracts of yeast strain Y206 (wildtype BY4741) were prepared in buffer AG200 as described in materials and methods. Purified recombinant MNase was added in a concentration of 0.5U per µg yeast protein (lanes 1–5) or in a concentration of 0.2U per µg yeast protein (lanes 6–10). Calcium chloride was added to a final concentration of 7 mM and the extracts were incubated at room temperature (22°C). Samples were taken after 0 minutes (lanes 1 and 6), after 5 minutes (lanes 2 and 7), after 10 minutes (lanes 3 and 8), after 20 minutes (lanes 4 and 9) and after 40 minutes (lanes 5 and 10) of incubation times. Total RNA was extracted and analyzed by agarose gel electrophoresis (upper panel) or by denaturing TBE/polyacrylamide gel electrophoresis (lower panel) followed by ethidium bromide staining as described in materials and methods. 25S rRNA, 18S rRNA, 5.8S rRNA, 5S rRNA and tRNA visible in lanes 1 and 6 are labeled on the left. (PDF) [file pone.0042449.s008.pdf]

A

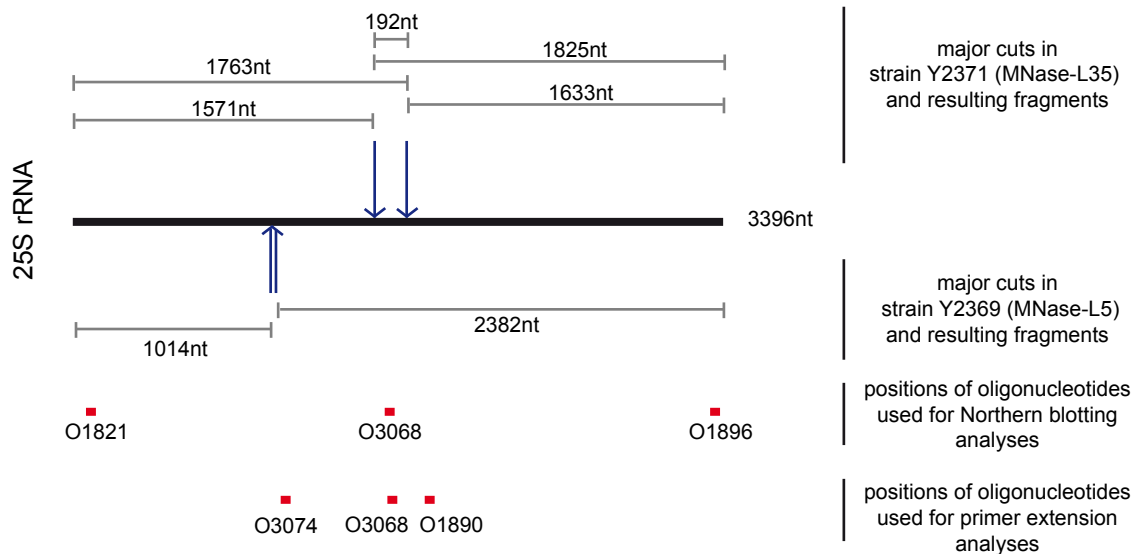

B

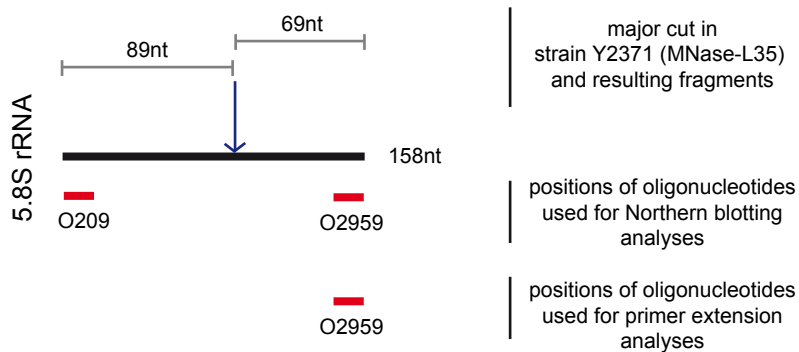

Supplement: Figure S9 — Map of 25S rRNA and 5.8S rRNA including positions of oligonucleotides used in this study and the major cuts observed in yeast strains Y2371 and Y2369. Oligonucleotides are represented by red boxes, major cleavage events by blue arrows and resulting rRNA fragments by grey lines. (PDF) [file pone.0042449.s009.pdf]
